# Effect of E-Beam Irradiation on Thermal and Mechanical Properties of Ester Elastomers Containing Multifunctional Alcohols

**DOI:** 10.3390/polym12051043

**Published:** 2020-05-02

**Authors:** Marta Piątek-Hnat, Kuba Bomba, Jakub Pęksiński, Agnieszka Kozłowska, Jacek G. Sośnicki, Tomasz J. Idzik

**Affiliations:** 1 Faculty of Chemical Technology and Engineering, West Pomeranian University of Technology, Piastów Ave. 42, 71-065 Szczecin, Poland; bk34688@zut.edu.pl (K.B.); agak@zut.edu.pl (A.K.); 2 Faculty of Electrical Engineering, West Pomeranian University of Technology, Sikorskiego Ave. 37, 71-313 Szczecin, Poland; jakub.peksinski@zut.edu.pl; 3 Department of Organic and Physical Chemistry, Faculty of Chemical Technology and Engineering, West Pomeranian University of Technology, Piastów Ave. 42, 71-065 Szczecin, Poland; jacek.sosnicki@zut.edu.pl (J.G.S.); tomasz.idzik@zut.edu.pl (T.J.I.)

**Keywords:** ester elastomers, sugar alcohols, e-beam irradiation, mechanical and thermal properties

## Abstract

The aim of this work was to investigate the thermal and mechanical properties of novel, electron beam-modified ester elastomers containing multifunctional alcohols. Polymers tested in this work consist of two blocks: sebacic acid–butylene glycol block and sebacic acid–sugar alcohol block. Different sugar alcohols were utilized in the polymer synthesis: glycerol, sorbitol, xylitol, erythritol, and mannitol. The polymers have undergone an irradiation procedure. The materials were irradiated with doses of 50 kGy, 100 kGy, and 150 kGy. The expected effect of using ionizing radiation was crosslinking process and improvement of the mechanical properties. Additionally, a beneficial side effect of the irradiation process is sterilization of the affected materials. It is also worth noting that the materials described in this paper do not require either sensitizers or cross-linking agent in order to perform radiation modification. Radiation-modified poly(polyol sebacate-co-butylene sebacate) elastomers have been characterized in respect to the mechanical properties (quasi-static tensile tests), cross-link density, thermal properties (Differential Scanning Calorimetry (DSC)), chemical properties: Fourier transform infrared spectroscopy (FTIR), and wettability (water contact angle). Poly(polyol sebacate-co-butylene sebacate) preopolymers were characterized with nuclear magnetic resonance spectroscopy (^1^H NMR and ^13^C NMR) and gel permeation chromatography (GPC). Thermal stability of cross-linked materials (directly after synthesis process) was tested with thermogravimetric analysis (TGA).

## 1. Introduction

Radiation modification is a method of cross-linking polymer materials that allows not only to save energy compared to a chemical cross-linking, but also provides a higher degree of control over the cross-link density. Some materials such as polyethylene are susceptible to radiation modification on their own; others, like polyisobutylene, require addition of a cross-linking agent. On the industrial scale, radiation cross-linking is used mostly in the production of poly(vinyl chloride) (PVC) and polyethylene (PE) wires and cables [1,2]. Other applications include modification of polyurethanes [3,4], polyamide 6 [5,6] and polyamide 12 [7], and rubber vulcanization [8,9].

Radiation cross-linking of polyesters in particular has attracted a noticeable amount of attention from researchers. Examples of aromatic polyesters cross-linked by radiation are poly(butylene terephthalate) (PBT) [10] and pol(ethylene terephthalate) (PET), though due to its properties it requires addition of a sensitizer [11,12]. Polyester-based thermoplastic elastomers can also be modified by radiation [13]

In case of biodegradable aliphatic polyesters, polylactide (PLA) have been most extensively tested. It has been radiation-modified with the use of triallyl isocyanurate as cross-linking agent [14,15,16]. Using pentaerythritol tetraacrylate as a cross-linking agent in order to modify both PLA and its copolymer, PLGA has also been reported [17]. It has also been reported that PLA cross-linking is possible without any crosslinking agents [18]. Other aliphatic polyesters cross-linked with radiation include polycaprolactone (PCl) cross-linked both with [19] and without [20] any cross-linking agents, and poly(butylene succinate) (PBS) in the presence of trimethallyl isocyanurate [21]

Electron-beam irradiation of sugar alcohol-based polyester elastomers has so far only been conducted in order to sterilize poly(glycerol sebacate) [22], even though this group of materials has been widely described in the literature.

Poly(glycerol sebacate) is the most notable example of sugar alcohol-based polyesters, and has been extensively investigated in respect of possible medical uses [23,24,25,26,27,28,29].

In general, sugar alcohol-based elastomers have been determined to be biocompatible and biodegradable [30,31]. A great advantage of those elastomers is the possibility of tailoring their properties by utilizing different sugar alcohols [32,33], or different dicarboxylic acids [34]. Third monomer, a diol can also be introduced into the synthesis, which leads to obtaining a material with better mechanical properties which can be fine-tuned by utilizing different diols [35]. Sugar alcohol-based copolyesters obtained using three monomers have also been described in our previous work [36,37,38].

Considering the many advantages of such polyesters we have concluded that the possibility of improving their properties even further by utilizing electron-beam radiation was a subject worth investigating. It is worth noting that utilizing radiation not only improves their properties, but also sterilizes the materials. The novelty in this work is that such modification of sugar alcohol-based elastomers synthesized using three monomers has never been described in the literature. 

## 2. Materials and Methods

### 2.1. Synthesis of Elastomers

All reagents were purchased from Sigma-Aldrich (St. Louis, MO, USA). Five polymers were synthesized utilizing sebacic acid, butanediol, and 5 different sugar alcohols (glycerol, erythritol, xylitol, sorbitol and mannitol). The monomer ratio of sebacic acid:sugar alcohol:butylene glycol was 2:1:1. Following materials were obtained: poly(glycerol sebacate-co-butylene sebacate) (PGBS), poly(erythritol sebacate-co-butylene sebacate) (PEBS), poly(xylitol sebacate-co-butylene sebacate) (PXBS), poly(sorbitol sebacate-co-butylene sebacate) (PSBS), and poly(mannitol sebacate-co-butylene sebacate) (PMBS).

The first step of each synthesis was esterification of sebacic acid, butylene glycol, and polyol in N_2_ atmosphere in 150 °C for 13.5 h. A flask with reaction mixture was heated in an oil bath until the substrates melted. Then, 3 mL of Ti(BuO)_4_ catalyst was added. The second step was polycondensation. Another portion of the catalyst, 2 mL of Ti(BuO)_4_, was added at the beginning of the polycondensation, which was conducted in a vacuum atmosphere in 150 °C for 3.5 h. The materials were then casted into silicone forms and were cross-linked in a vacuum dryer in 100 °C in 100 mb. 

### 2.2. Irradiation

Materials after cross-linking were e-beam irradiated in the Institute of Nuclear Chemistry and Technology (Warsaw) A linear electron accelerator Elektronika 10/10 (NPO Torij, Russia) was used to generate a 10 MeV beam of different dosages, namely, 50, 100, and 150 kGy. Radiation was split into 25 kGy doses. Average set current was 360 mA; samples were moved with 0.368 m/min speed. The process was carried out in adherence to PN-ISO 11137-2007 standard.

### 2.3. Experimental Methods

#### 2.3.1. Nuclear Magnetic Resonance Spectroscopy (NMR)

^1^H and ^13^C NMR spectroscopic measurements were performed on a Bruker DPX 400 AVANCE III HD spectrometer (Bruker, Rheinstetten, Germany) operating at 400.1 and 100.6 MHz, respectively. Approximately 50 mg of each sample was dissolved in 0.7 mL of deuterated chloroform (CDCl_3_). TMS was used as internal reference and spectra were acquired in 5 mm probes. For NMR analyses, MestReNova (version 12.0.3, Mestrelab, Santiago de Compostela, Spain) program was used.

#### 2.3.2. Fourier Transform Infrared Spectroscopy (FTIR)

Analysis of the chemical structure of the polymers was conducted with Fourier transform infrared spectroscopy (FTIR). An Alpha Spectrometer Bruker (Bruker, Germany) was used. Recorded transmission spectra were in the range between 4000 cm^−1^ and 400 cm^−1^ with resolution of 2 cm^−1^. In order to develop the results, Omnic 7.3 software by the Thermo Electron Corporation (Waltham, MA, USA) was used. Analyses were performed on elastomers before and after irradiation.

#### 2.3.3. Differential Scanning Calorimetry (DSC)

In order to determine the thermal properties of the materials, differential scanning calorimetry (DSC) was utilized. TA Instruments apparatus Q2500 (New Castle, DE, USA) was used. Parameters of the analysis were −100 °C to 200 °C heating cycle and 10 °C/min heating rate. Tests were performed in a nitrogen atmosphere. In order to develop the results, TA Instruments Universal Analysis 2000, 3.9a software (New Castle, DE, USA) was used. Tests were performed on elastomers before and after irradiation.

#### 2.3.4. Thermogravimetric Analysis (TGA)

TGA analysis was performed in order to analyze the thermal stability of the elastomers. Q500 TGA instrument (TA Instruments, New Castle, DE, USA) equipped with platinum crucibles was used. A heating rate of 2 °C was utilized. The temperature range was 25 °C to 600 °C. Weight of the samples was ~15 mg. The test was conducted in dry air atmosphere. 

Analysis was performed for non-irradiated, cross-linked elastomer samples, taken directly after synthesis. 

#### 2.3.5. Mechanical Properties

Testing of the mechanical properties was performed using Instron 36 instrument (Norwood, MA, USA). Parameters of the tests were 25 °C, 50% of relative humidity, 100 mm/min crosshead speed, and 500 N load cell. Tests were performed in keeping with PN-EN-ISO 526/1:1996 standard. Tests were performed on elastomers before and after irradiation

#### 2.3.6. Water Contact Angle

Measurement of the water contact angle was carried out with KRUSS DSA100 digital goniometer (Hamburg, Germany). In order to perform static contact angle tests, 2 µL droplet of deionized water was placed on the surface of degreased materials. Automatic dispenser was used. Calculation of the contact angle was carried out with drop shape analysis software (DSA4) by Kruss (Hamburg, Germany). Tests were performed on elastomers before and after irradiation.

#### 2.3.7. Cross-Link Density

Two grams of polymer samples of each material before and after irradiation was prepared. Three samples of each material were prepared. Each sample was immersed in 20 mL of Tetrahydrofuran (THF) in 20 °C for 5 days. After that, polymer samples were separated from the solvent and weighed (wet fraction). Next, polymer samples were dried for 8 days in a vacuum dryer in 20 °C and weighed again (dry fraction). 

The Flory–Rehner equation [39] was used to calculate the cross-link density,

(1) $$\nu =\frac{\ln\left(1-\upsilon_{2}\right)+\upsilon_{2}+\chi \upsilon_{2}^{2}}{\upsilon_{1}\left(\left(\frac{\upsilon_{2}}{2}\right)-\upsilon_{2}^{\frac{1}{3}}\right)}$$

(2) $$\upsilon_{2}={\left[1+\left(\frac{m_{1}-m_{2}}{m_{2}}\right)\;\left(\frac{\rho_{s}}{\rho_{p}}\right)\right]}^{-1}$$

where *v* is the strand density (half of the cross-link density), *v*_2_ is the polymer volume fraction at equilibrium swelling, χ is the polymer–solvent interaction parameter (*χ* = 0.42), $\rho_{s}$ is the solvent density, $\rho_{p}$ is the polymer density, *v*_1_ is the solvent molar volume, *v*_2_ is the polymer volume fraction at equilibrium swelling, *m*_1_ is the wet fraction weight, and *m*_2_ is the dry fraction weight

#### 2.3.8. Gel Permeation Chromatography (GPC)

Determination of the molecular weights of the PGBS, PEBS, PXBS, PSBS, and PMBS prepolymers was conducted using gel permeation chromatography (GPC). Styragel column (Waters, Milford, MA, USA) was utilized. Samples were dissolved (1 mg/mL) in tetrahydrofuran (THF). 

## 3. Results and Discussion

**Figure 1:**
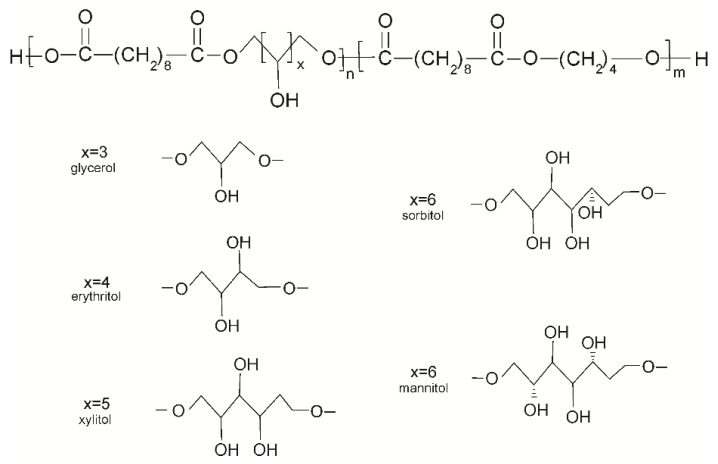
Scheme of of poly(polyol sebacate-co-butylene sebacate) structure.

The composition and properties of elastomers are summarized in Table 1 and Table 2, and a scheme of the structure is shown in Figure 1. 

### 3.1. Nuclear Magnetic Resonance Spectroscopy (NMR)

In order to confirm the success of the performed syntheses and establish the chemical structure of the obtained polymers, both ^13^C NMR and ^1^H NMR were performed.

**Figure 2:**
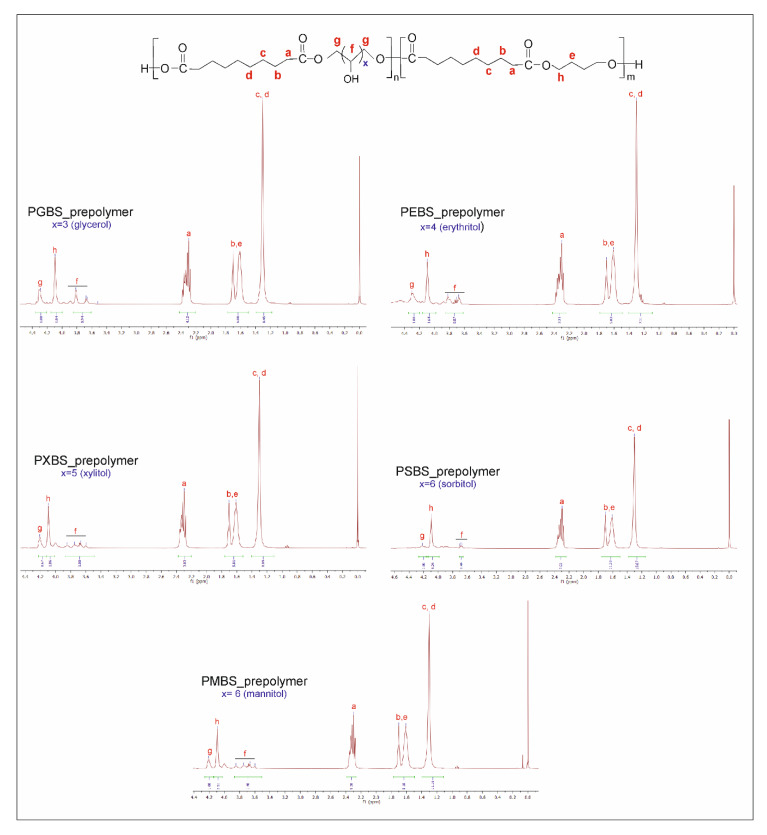
Nuclear magnetic resonance spectroscopy (^1^H NMR) of poly(glycerol sebacate-co-butylene sebacate) (PGBS), poly(erythritol sebacate-co-butylene sebacate) (PEBS), poly(xylitol sebacate-co-butylene sebacate) (PXBS), poly(sorbitol sebacate-co-butylene sebacate) (PSBS), and poly(mannitol sebacate-co-butylene sebacate) (PMBS) prepolymers.

In ^1^H NMR (Figure 2) two peaks assigned to a proton next to an ester bond can be seen: the peak at ~4.25 ppm corresponds to a proton next to an ester bond between sugar alcohol and sebacic acid, and the peak at ~4.1 ppm corresponds to an ester bond between butylene glycol and sebacic acid. 

The ratio of the area of the peak at 4.25 ppm to the area of the peak at 4.1 ppm tells us the reactivity of sugar alcohols as compared to butylene glycol. Erythritol is the most reactive, with the ratio of sebacic acid–butylene glycol ester bonds to sebacic acid–sugar alcohol ester bonds being 1.64, while sorbitol is the least reactive, with a 4.24 ratio. Those results correspond well to the M_w_ of the polymers, determined by GPC.

The peak at 1.30 ppm is connected to CH_2_ (c) and CH_2_ (d) groups in sebacic acid, and the doublet at about 1.61 and 1.70 ppm is linked to CH_2_ (b) group in sebacic acid and CH_2_ (e) group in butylene glycol. The peak at 2.30 ppm is linked to CH_2_ (a) groups in sebacic acid. The peaks between 3.60 and 3.85 ppm are connected to hydroxyl groups in sugar alcohols.

**Figure 3:**
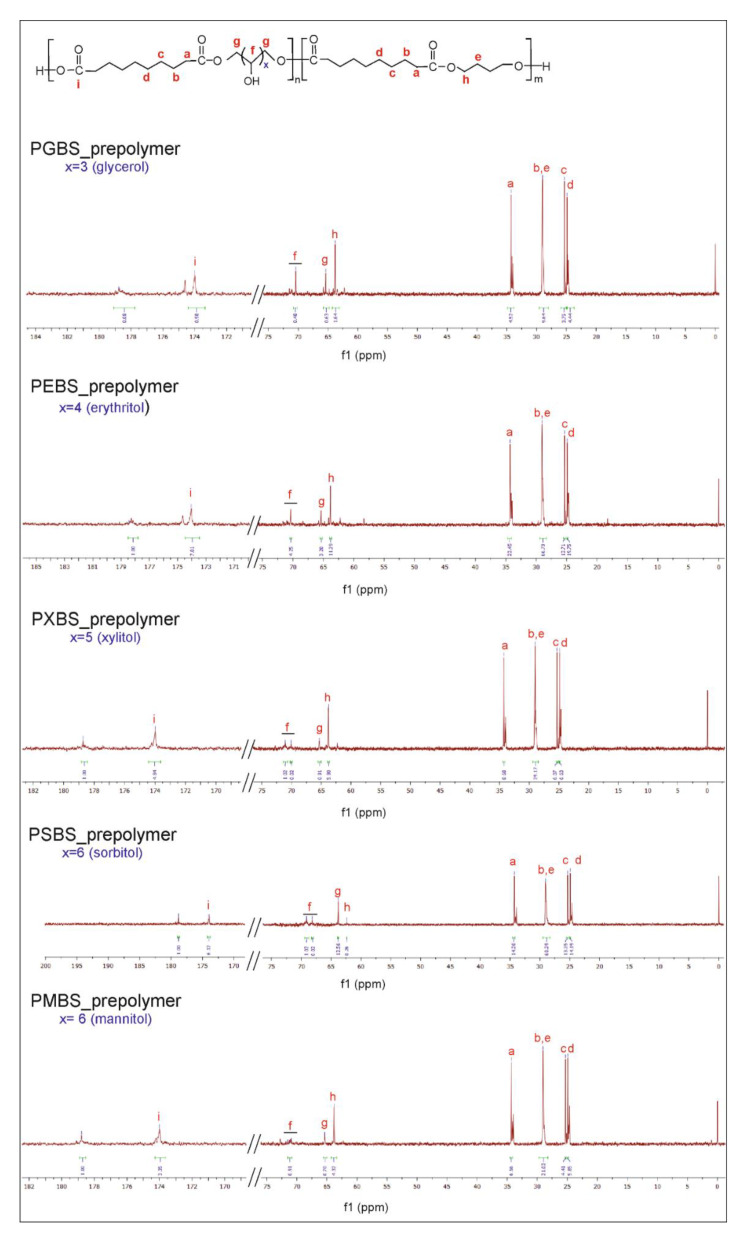
^13^C NMR of PGBS, PEBS, PXBS, PSBS, and PMBS prepolymers.

In ^13^C NMR (Figure 3), two peaks assigned to carbons next to an ester bond: the peak at ~63.8 ppm corresponds to a C(h) carbon next to an ester bond between butylene glycol and sebacic acid, and the peak at ~65.3 ppm corresponds to a C(g) carbon next to an ester bond between sugar alcohol and sebacic acid. Peaks in the 70 to 72 ppm range are due to carbon atoms in CH_2_OH groups in sugar alcohols. The peak at 174 ppm is connected to C(i) carbon atoms in C=O groups. The peak at 178 ppm is due to –COOH groups in leftover unreacted carboxylic acid.

The peak at ~24.9 ppm is linked to CH_2_ (d) group, peak at about 25.3 ppm is connected to CH_2_ (c) group, and the peak at about 29.00 ppm was assigned to both the CH_2_ (b) group in sebacic acid and CH_2_ (e) group in butylene glycol. Peak at about 34.3 ppm was ascribed to CH_2_ (a) group in sebacic acid. 

### 3.2. Fourier Transform Infrared Spectroscopy (FTIR)

Four transmittance peaks characteristic for sugar alcohol-based polyesters can be seen on the FTIR spectra (Figure S1–S5).

The peak at 1170 cm^−1^ is related to –C–O–C groups, the peak at 2930 cm^−1^ is linked to –CH groups, the peak at 3450 cm^−1^ is associated with –OH groups, and the peak at 1730 cm^−1^ was assigned to –C=O groups. Radiation modification did not cause any significant changes in the intensity of peaks connected to –CH- and –C=O groups, but a slight increase in the intensity of peaks assigned to –C–O–C groups can be seen, due to the cross-linking process taking place. In the spectra of PEBS, PGBS, and PMBS, peak associated with –OH groups split into two. Peak emerging at about 3200 cm^−1^ is ascribed to –OH groups that are not hydrogen-bound. It is a result of hydrogen bonds between –OH groups in neighboring polymer chains breaking apart due to irradiation. This process is most strongly visible in case of PEBS material. It leads to elongation at break being lower in the irradiated material than in the not irradiated material.

### 3.3. Thermal Properties: Differential Scanning Calorimetry (DSC) and Thermogravimetric Analysis (TGA)

**Figure 4:**
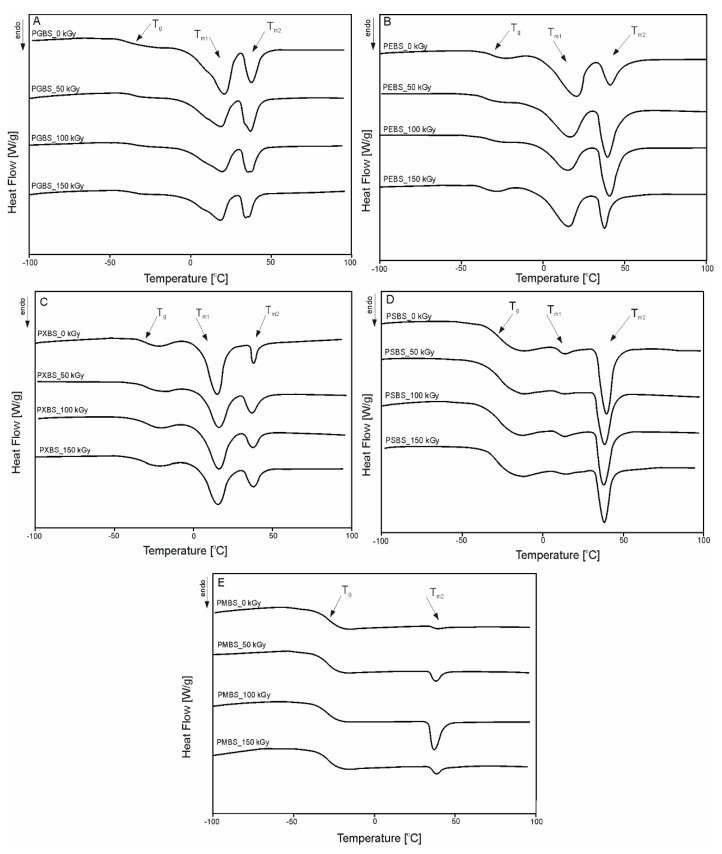
First-heating differential scanning calorimetry (DSC) thermograms of (**A**) PGBS, (**B**) PEBS, (**C**) PXBS, (**D**) PSBS, and (**E**) PMBS before and after irradiation.

The DSC 1nd heating thermograms for PXBS, PSBS, PEBS, PGBS, and PMBS are shown in Figure 4, and the thermal properties of the elastomers before and after irradiation are given in Table 3.

All materials expect PMBS exhibit two melting transitions. *T*_m1_ is attributed to the melting of the sebacic acid−polyol blocks, and *T*_m2_ is a result of melting of the sebacic acid−butanediol blocks.

In the first heating, all the materials affected by e-beam radiation exhibit some changes in their transition temperatures and enthalpies. Temperature values associated with glass transition exhibit only small alterations between non-irradiated materials and irradiated materials However, a noticeable change in Δ*C*_p_, can be observed. It is linked to the changes in the amorphous phase and rearrangement of the cross-linked structure caused by irradiation process. Those changes are connected with the alteration of the mechanical properties and cross-link density of the materials shown by the mechanical tests, and cross-link density results

**Figure 5:**
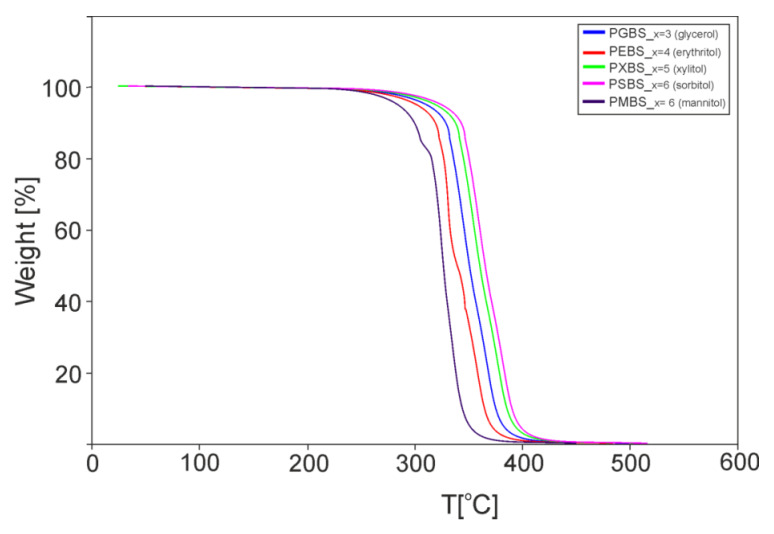
Thermogravimetric analysis (TGA) of the cross-linked samples taken directly after synthesis.

A noticeable decrease in *T*_m1_ of PEBS after radiation modification of the material is linked to the breaking apart of the hydrogen bonds between erythritol particles in adjacent polymer chains which was confirmed by the FTIR analysis. TGA results for cross-linked samples taken directly after synthesis are shown in Figure 5. All materials show similar slopes of the function curve, and have thermal stability up to 220 °C.

### 3.4. Mechanical Properties

**Figure 6:**
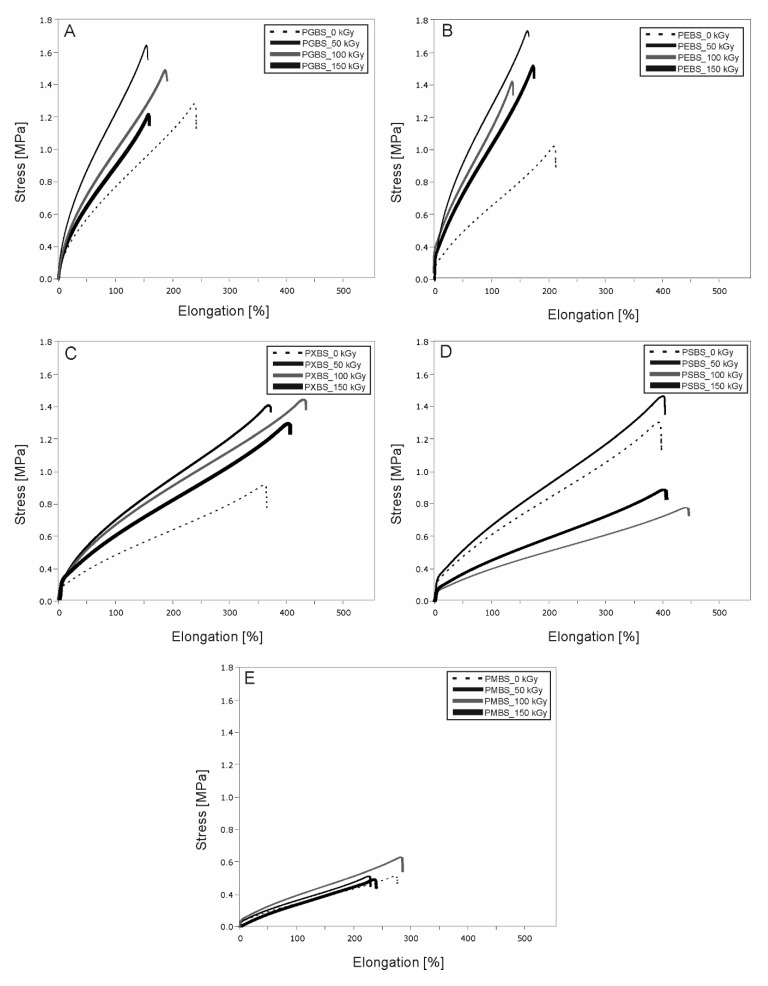
Mechanical properties of (**A**) PGBS, (**B**) PEBS, (**C**) PXBS, (**D**) PSBS, and (**E**) PMBS before and after irradiation.

Figure 6 shows the results of the mechanical tests of the elastomers before and after radiation modification. For all the non-irradiated samples except PSBS, values of the moduli at 100% and 50% elongation (Table 1) decrease with the increase of hydroxyl group content.

In general, a noticeable change of the mechanical properties of radiation-modified materials can be observed. PGBS and PEBS seem to be most receptive to the procedure, with all of their mechanical properties improving. PMBS material is the least receptive to the radiation modification, and should not be modified in such way. The 50 kGy dose leads to the greatest improvement of mechanical properties, whereas the effects caused by the 150 kGy dose are the least desirable. Therefore, modifying such materials with 50 kGy radiation can be considered the best choice.

### 3.5. Cross-Link Density

**Figure 7:**
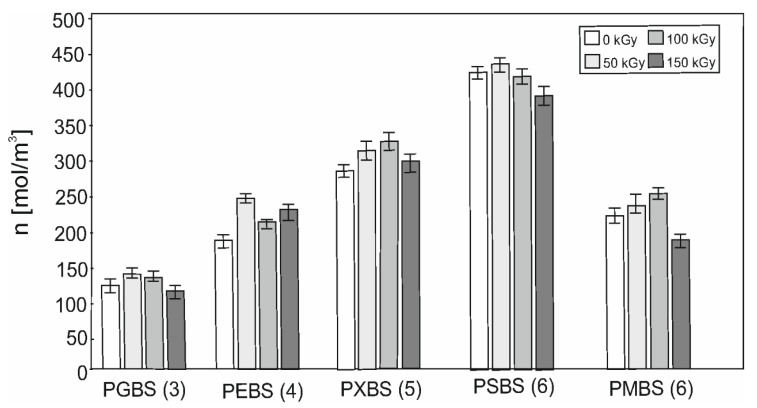
Crosslinking density of PGBS, PEBS, PXBS, PSBS, and PMBS before and after irradiation. Numbers in the brackets indicate the amount of hydroxyl groups in the sugar alcohols.

Cross-link density (Figure 7) of materials before irradiation depends on the sugar alcohol used for the synthesis. It can be observed that the cross-link density increases with increasing amount of hydroxyl groups in sugar−alcohols, except for mannitol, in which not all hydroxyl groups take part in the cross-linking reaction due to its stereochemical structure. In all cases, the 50 kGy dose seems to be the most optimal and leads to increase of cross-link density, which directly correlates with improved mechanical properties. 150 kGy dose leads to the least desirable results and should not be used to modify those materials.

### 3.6. Water Contact Angle

**Figure 8:**
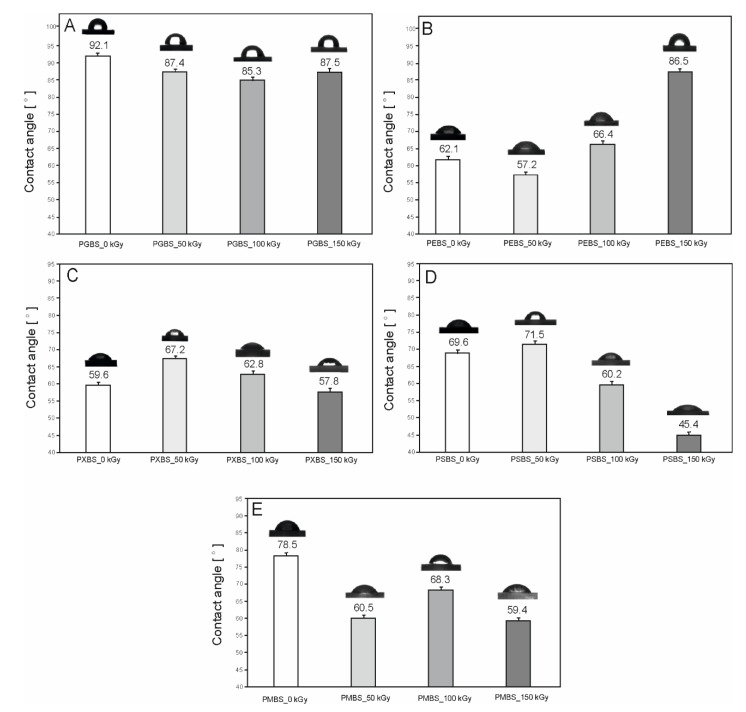
Contact angle of (**A**) PGBS, (**B**) PEBS, (**C**) PXBS, (**D**) PSBS, and (**E**) PMBS before and after irradiation.

Irradiation of the elastomers leads to a change in the contact angle for all materials. It can be observed that the improvement of mechanical properties does not correlate to the enhancement of their hydrophilic characteristics (Figure 8).

While in the case of PXBS and PSBS a 50 kGy dose shows the best results, which is a similar tendency to that observed in the mechanical properties section of the paper, in the case of the PEBS elastomer, 150 kGy leads to the most noteworthy improvement. This may be linked to the increase of non-hydrogen-bonded –OH groups being present, which is confirmed by the FTIR analysis. For PGBS and PMBS elastomers, radiation modification actually worsens their hydrophilic properties, with the decline being most apparent for PMBS material, which reinforces our conclusion that it should not be modified in such a way. Improved wettability leads to better cell adhesion, which is important for eventual future biomedical uses.

## 4. Conclusions

The effects of radiation modification of sugar−alcohol-based polyesters were studied. Chemical, thermal, and mechanicals properties were all affected. Materials affected by 50 kGy dose showed the greatest improvement, whereas treatment with a 150 kGy dose leads to least desirable properties. Selection of the right dose of radiation is important for possible future industrial applications. A change in the amorphous structure confirmed by DSC was also apparent. All materials except PMBS were concluded to be susceptible and well suited for such modification, as confirmed by the improvement of mechanical properties and the increase of cross-link density. Radiation modification has the advantage not only because of the improvement of the mechanical properties, but also because affected materials are sterilized. Simplicity of modification of such materials is worth noting: no cross-linking agents or sensitizers are required, and the materials can be modified directly after synthesis, without any additional processing.

## Figures and Tables

**Table 1:** Composition and molecular weight distributions of pre-polymers. Poly(glycerol sebacate-co-butylene sebacate) (PGBS), poly(erythritol sebacate-co-butylene sebacate) (PEBS), poly(xylitol sebacate-co-butylene sebacate) (PXBS), poly(sorbitol sebacate-co-butylene sebacate) (PSBS), and poly(mannitol sebacate-co-butylene sebacate) (PMBS).

Pre-Polymer	Molar Composition [mol]			*M*_w_ [g/mol]	PDI	Composition (PBS to PPS Segments) by ^1^H NMR
PGBS	SA	GL	BG	40,000	2.1	1.84:1
	2	1	1			
PEBS	SA	ER	BG	42,000	1.7	1.64:1
	2	1	1			
PXBS	SA	XL	BG	38,000	1.8	1.86:0.67
	2	1	1			
PSBS	SA	SB	BG	20,000	1.4	4.24:1
	2	1	1			
PMBS	SA	MN	BG	29,000	2.5	2.51:1
	2	1	1			

where *M*_w_: weight average molecular weight, PDI: polydispersity index, SA: sebacic acid, BG: butylene glycol, XL: xylitol, SB: sorbitol, ER: erythrytol, GL: glycerol, Mn: mannitol, PBS: poly(butylene sebacate) segment, PPS: poly(polyol sebacate) segment.

**Table 2:** Composition and selected properties of poly(glycerol sebacate-co-butylene sebacate) (PGBS), poly(erythritol sebacate-co-butylene sebacate) (PEBS), poly(xylitol sebacate-co-butylene sebacate) PXBS, poly(sorbitol sebacate-co-butylene sebacate) (PSBS),poly(mannitol sebacate-co-butylene sebacate) (PMBS) before and after irradiation.

Material/Dose	Molar Composition [mol]			E_ 50% [MPa]	E_ 100% [MPa]	σ_r_ [MPa]	ε_r_ [%]	*n* [mol/m^3^]
-	SA	GL	BG	-				
PGBS_0 kGy	2	1	1	1.34 +/−0.09	0.910 +/−0.04	1.34 +/−0.35	230 +/−92.66	120.45 +/−52.66
PGBS_50 kGy				2.77 +/−0.69	1.50 +/−0.35	1.62 +/−0.37	156 +/−47.44	142.31 +/−43.32
PGBS_100 kGy				2.44 +/−0.36	1.34 +/−0.15	1.55 +/−0.26	186 +/−59.41	139.20 +/−57.21
PGBS_150 kGy				1.95 +/−0.38	1.13 +/−0.16	1.22 +/−0.21	143 +/−30.86	110.42 +/−28.81
-	SA	ER	BG	-				
PEBS_0 kGy	2	1	1	0.799 +/−0.16	0.632 +/−0.09	1.06 +/−0.35	219 +/−85.12	189.58 +/−75.22
PEBS _50 kGy				2.84 +/−0.76	1.60 +/−0.33	1.75 +/−0.36	163 +/−40.65	249.23 +/−53.71
PEBS_100 kGy				2.06 +/−0.45	1.36 +/−0.15	1.38 +/−0.33	139 +/−47.89	215.32 +/−43.59
PEBS_150 kGy				2.33 +/−0.58	1.38 +/−0.20	1.56 +/−0.23	172 +/−43.60	231.42 +/−46.88
-	SA	XL	BG	-				
PXBS_0 kGy	2	1	1	0.345 +/−0.04	0.296 +/−0.04	0.931 +/−0.46	362 +/−68.8	287.42 +/−57.32
PXBS_50 kGy				0.492 +/−0.08	0.424 +/−0.07	1.41 +/−0.35	372 +/−52.3	310.58 +/−45.24
PXBS_100 kGy				0.456 +/−0.14	0.395 +/−0.11	1.47 +/−0.47	431 +/−81,13	321.44 +/−41.54
PXBS_150 kGy				0.345 +/−0.03	0.298 +/−0.03	1.29 +/−0.22	409 +/−32.91	299.32 +/−38.87
-	SA	SB	BG	-				
PSBS_0 kGy	2	1	1	0.536 +/−0.11	0.455 +/−0.03	1.32 +/−0.41	395 +/−37.08	430.15 +/−45.13
PSBS_50 kGy				0.826 +/−0.13	0.624 +/−0.07	1.49 +/−0.18	400 +/−17.50	437.11 +/−38.63
PSBS_100 kGy				0.234 +/−0.04	0.216 +/−0.03	0.77 +/−0.14	440 +/−57.21	425.38 +/−47.28
PSBS _150 kGy				0.311 +/−0.12	0.269 +/−0.09	0.89 +/−0.25	403 +/−33.90	387.31 +/−43.76
-	SA	MN	BG	-				
PMBS_0 kGy	2	1	1	0.329 +/−0.13	0.264 +/−0.08	0.593 +/−0.17	275 +/−44.70	221.32 +/−42.85
PMBS_50 kGy				0.394 +/−0.10	0.316 +/−0.06	0.557 +/−0.10	221 +/−23.87	241.48 +/−45.31
PMBS_100 kGy				0.384 +/−0.09	0.302 +/−0.06	0.629 +/−0.11	281 +/−30.51	254.32 +/−49.28
PMBS_150 kGy				0.347 +/−0.14	0.275 +/−0.08	0.489 +/−0.12	237 +/−29.77	189.41 +/−39.87

where: σ_r_: Stress in break, ε: Elongation, E_50%: Modulus at 50% elongation, E_100%: Modulus at 100% elongation, *n*: Cross-linking density, SA: sebacic acid, BG: butylene glycol, XL: xylitol, SB: sorbitol, ER: erythrytol, GL: glycerol, Mn: mannitol.

**Table 3:** DSC termograms of poly(glycerol sebacate-co-butylene sebacate) (PGBS), poly(erythrytol sebacate-co-butylene sebacate) (PEBS), poly(xylitol sebacate-co-butylene sebacate) PXBS, poly(sorbitol sebacate-co-butylene sebacate) (PSBS), poly(mannitol sebacate-co-butylene sebacate) (PMBS) before and after irradiation.

Material/Dose	I HEATING					
	*T*_g_	Δ*C*_p_	*T*_m1_	Δ*H*_m1_	*T*_m2_	Δ*H*_m2_
	[°C]	[J/g°C]	[°C]	[J/g]	[°C]	[J/g]
PGBS	-					
PGBS_0 kGy	−39.4	0.508	21.1	48.9	38.2	17.1
PGBS_50 kGy	−36.1	0.319	18.9	35	37.2	18.7
PGBS _100 kGy	−36.4	0.276	19.9	30.5	37.9	14.4
PGBS _150 kGy	−37.5	0.216	18.6	31.2	34.2	12.1
PEBS	-					
PEBS_0 kGy	−36.5	0.248	20.2	28.8	41.6	10.6
PEBS_50 kGy	−35.9	0.341	16.4	24.6	39.6	22.2
PEBS_100 kGy	−33.4	0.259	14.4	19.9	40.9	24.4
PEBS_150 kGy	−37.4	0.267	14.9	26.9	37.4	10.5
PXBS	-					
PXBS_0 kGy	−31.6	0.368	15.3	30.6	38.4	3.8
PXBS_50 kGy	−30.9	0.387	16.6	21.5	37.4	6.7
PXBS_100 kGy	−34.1	0.423	16.2	24.9	38.3	5.1
PXBS_150 kGy	−33.5	0.39	15.6	23.9	38.3	6.5
PSBS	-					
PSBS_0 kGy	−29.2	0.562	14.4	1.2	39.7	12.7
PSBS_50 kGy	−29.7	0.646	13.9	0.485	38.6	10.6
PSBS_100 kGy	−29.7	0.633	13.9	1.1	38.3	10.5
PSBS_150 kGy	−29.2	0.6	14.3	0.543	38.7	9.5
PMBS	-					
PMBS_0 kGy	−30.1	0.69	−	−	39.8	0.56
PMBS_50 kGy	−30.9	0.726	−	−	38.4	2.3
PMBS _100 kGy	−30.2	0.627	−	−	37	6,8
PMBS_150 kGy	−29.7	0.64	−	−	38.7	1.5

where ∆*C*_p_: change of the heat capacity_,_, *T*_g_: glass transition temperature, *T_m_*_1_ and *T_m_*_2_: melting temperature, *∆H_m_*_1_ and *∆H_m_*_2_: heat of melting.

## Supplementary Materials

The following are available online at https://www.mdpi.com/2073-4360/12/5/1043/s1, Figure S1: FTIR spectra of poly(glycerol sebacate-co-butylene sebacate) (PGBS) before and after irradiation, Figure S2: FTIR spectra of poly(erythrytol sebacate-co-butylene sebacate) (PEBS) before and after irradiation, Figure S3: FTIR spectra of poly(xylitol sebacate-co-butylene sebacate) (PXBS) before and after irradiation, Figure S4: FTIR spectra of poly(sorbitol sebacate-co-butylene sebacate) (PSBS) before and after irradiation, Figure S5: FTIR spectra of poly(mannitol sebacate-co-butylene sebacate) (PMBS) before and after irradiation.
