# Supplementary material for: Effect of E-Beam Irradiation on Thermal and Mechanical Properties of Ester Elastomers Containing Multifunctional Alcohols
Source: Polymers (Basel). 2020 May 2;12(5):1043. doi: 10.3390/polym12051043 (PMC7284812; doi:10.3390/polym12051043)
Supplement: Supplementary file 1 [file polymers-12-01043-s001.pdf]

Effect of E-Beam Irradiation on Thermal and Mechanical Properties of Ester Elastomers Containing Multifunctional Alcohols

Marta Piątek-Hnat, Kuba Bomba, Jakub Pęksiński and Agnieszka Kozłowska, Jacek G. Sośnicki, Tomasz J. Idzik

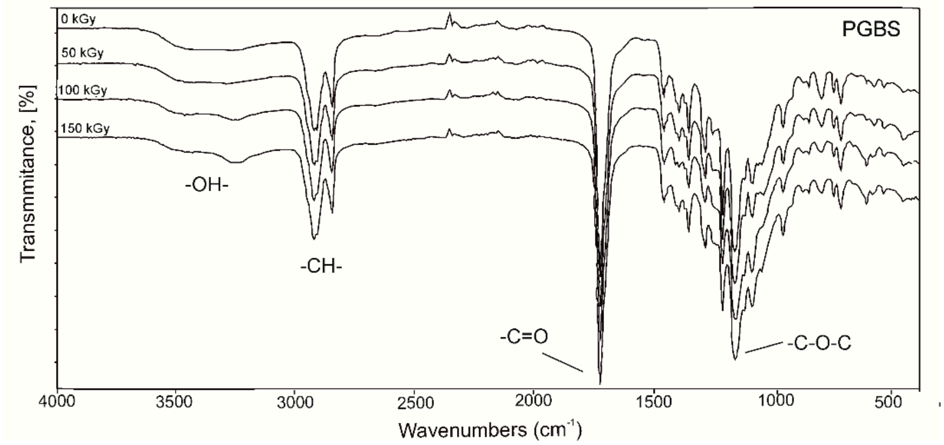

Figure S1. FTIR spectra of poly(glycerol sebacate-co-butylene sebacate) (PGBS) before and after irradiation.

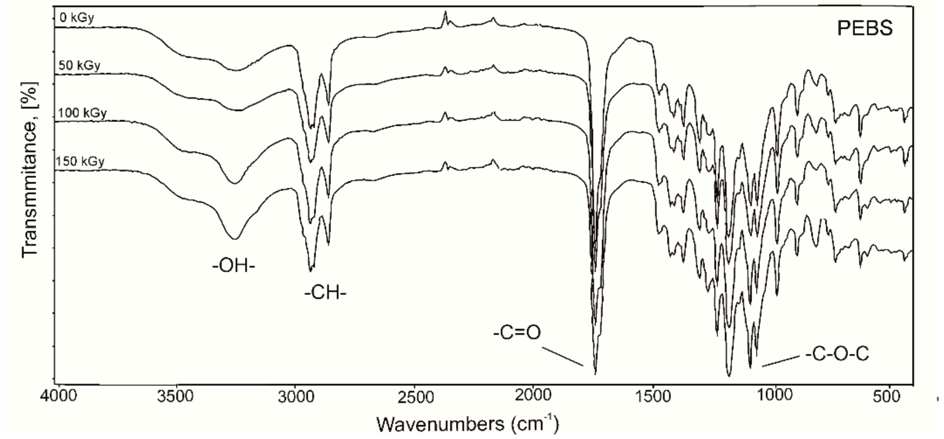

Figure S2. FTIR spectra of poly(erythrytol sebacate-co-butylene sebacate) (PEBS) before and after irradiation.

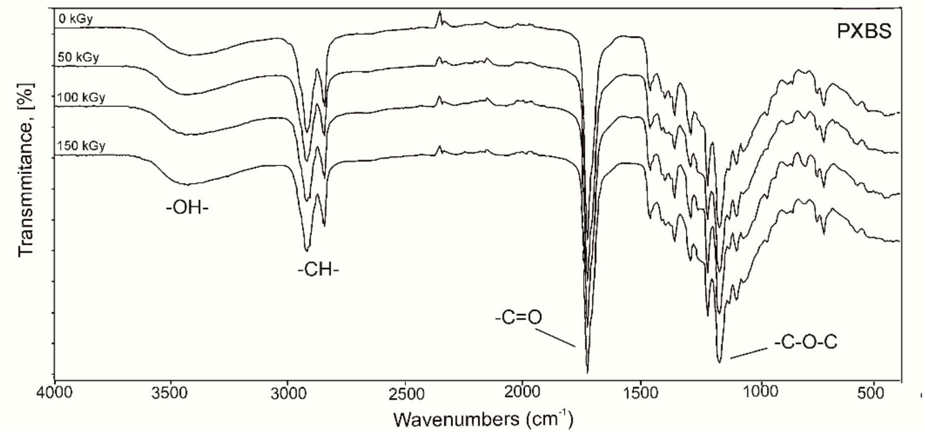

Figure S3. FTIR spectra of poly(xylitol sebacate-co-butylene sebacate) (PXBS) before and after irradiation.

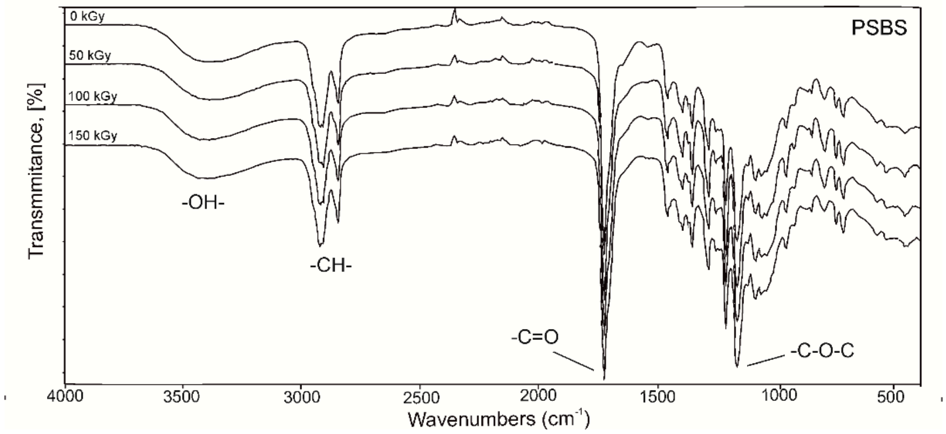

Figure S4. FTIR spectra of poly(sorbitol sebacate-co-butylene sebacate) (PSBS) before and after irradiation.

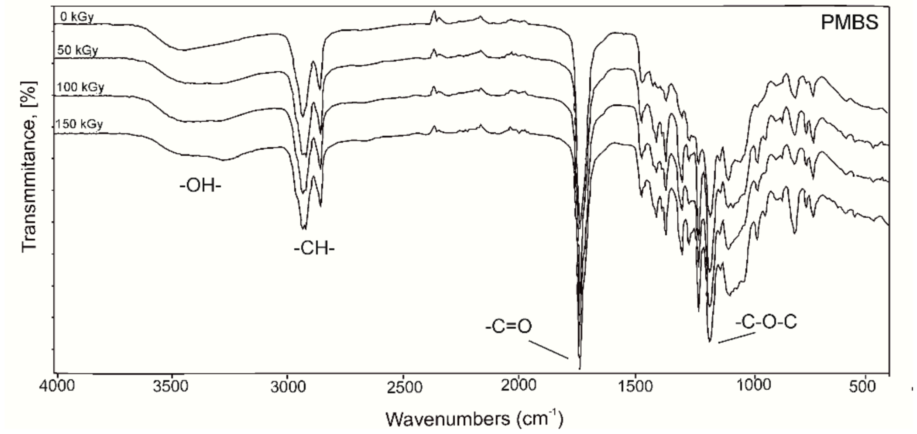

Figure S5. FTIR spectra of poly(mannitol sebacate-co-butylene sebacate) (PMBS) before and after irradiation.
